# Predominant negative symptoms: views of patients vs. doctors in a 1-year observational study

**DOI:** 10.1192/j.eurpsy.2024.379

**Published:** 2024-08-27

**Authors:** J. Dragasek, Z. B. Dombi, K. Acsai, V. Dzurilla, Á. Barabássy

**Affiliations:** ^1^University of P. J. Safarik, Kosice, Slovakia; ^2^Gedeon Richter Plc.; ^3^Ceva Animal Health, Budapest, Hungary; ^4^Gedeon Richter Slovakia, Bratislava, Slovakia

## Abstract

**Introduction:**

Negative symptoms are a key aspect of schizophrenia, significantly impacting a patient’s functioning and quality of life. These symptoms are deemed predominant when they dominate the clinical picture and positive symptoms are only minimally present. As articulated in the most recent guidance by the European Psychiatric Association, including self-report measures is encouraged in negative symptom studies as they can further complement the observer-rated scales when assessing negative symptoms of schizophrenia.

**Objectives:**

The objective of the poster is to compare the views of patients vs. doctors regarding predominant negative symptoms during a 1-year observational study.

**Methods:**

This was a 1-year-long, prospective, multicentric cohort study with three visits after baseline at 3, 6 and 12 months. Adult outpatients with a schizophrenia diagnosis according to the International Classification of Diseases 10th edition who exhibited predominant negative symptoms according to clinical judgement were included. Patients received pharmacological and some non-pharmacological treatment as usual.

The primary outcome measure was the modified Short Assessment of Negative Domains (m-SAND), an anamnesis-based scale that is composed of 7 items: two positive items (delusions and hallucinations) which make the m-SAND Positive sub-scale (m-SAND-P) and five negative items (anhedonia, alogia, avolition, asociality and affective flattening) which make the m-SAND Negative sub-scale (m-SAND-N) Each item is rated from 0 to 5 (not observed; mild; moderate; moderately severe; severe; and extreme). Other measurements included the Self-evaluation of Negative Symptoms (SNS), a validated scale that provides meaningful information regarding the patients’ own perception of their negative symptoms.

Least squares (LS) means were calculated for the change from baseline to final visit using a mixed model for repeated measures (MMRM).

**Results:**

188 patients were included in the study. The mean age was 39.8 years and 65% of patients were men. The mean duration of illness was 12 years. At baseline, patients rated alogia and apathy (mean SNS score: 5.7) to be the most severe and then asociality (5.5). In contrast, doctors found affective blunting (mean m-SAND total score: 4.3), apathy (4.2) and anhedonia (4.0) to be the most severe.

After the end of the observational period all negative symptom sub-domains improved significantly according to both the patients’ and doctors’ views. The latter group reported -1.9 LS mean change from baseline in apathy, -1.8 in anhedonia, and -1.7 in asociality (all p-value <0.0001). Patients felt most change in alogia and asociality (-2.7), and apathy and anhedonia (-2.4).

**Conclusions:**

In summary, both patients and doctors reported significant improvement in predominant negative symptoms. Nonetheless, there were some differences how they perceived severity and change in the specific domains.

**Disclosure of Interest:**

J. Dragasek: None Declared, Z. Dombi Employee of: Gedeon Richter Plc., K. Acsai: None Declared, V. Dzurilla Employee of: Gedeon Richter Plc., Á. Barabássy Employee of: Gedeon Richter Plc.

